# The protective effect of zinc oxide nanoparticles on boar sperm during preservation at 17 °C

**DOI:** 10.1590/1984-3143-AR2023-0013

**Published:** 2025-01-20

**Authors:** Qimeng Hu

**Affiliations:** 1 Hebei Key Laboratory of Animal Diversity, College of Life Sciences, Langfang Normal University, Hebei Langfang, China

**Keywords:** capacitation, sperm, protein, oxidative stress

## Abstract

More than 90% of spermatozoa of boars in pork producing countries is stored in liquid at 17 °C; however, the quality of these spermatozoa is affected by bacterial breeding and oxidative damage. This study analyzed sperm quality and sperm capacitation after storage to study the effects of the effects of ZnO nanoparticles (ZnO NPs) supplementation on seminal plasma (SP)-free sperm preservation. We investigated the effects of adding 20, 50, 100 and 200 μg/mL of ZnO NPs to a seminal free boar sperm diluent over a 7-day period at 17 °C to assess the changes in non-capacitated/capacitated sperm quality parameters, antioxidant capacity, ATP content and extent of protein tyrosine phosphorylation. The addition of different doses of ZnO NPs to stored sperm did not induce significant effects on the sperm motility and ATP content when compared to the sperm without ZnO NPs treatment. However, the addition of 50, 100, 200 μg/mL ZnO NPs to stored sperm improved total antioxidant capacity (T-AOC) and CuZn-superoxide dismutase (CuZn-SOD) (*p* < 0.05). ZnO NPs also reduced the malondialdehyde (MDA) content of the preserved sperm (*p* < 0.05). Moreover, our results indicate that the supplementation of 50 μg/mL ZnO NPs to preserved sperm improved the sperm membrane integrity (*p* < 0.05). ZnO NPs exerted protective effects on protein tyrosine phosphorylation, especially with regards to membrane proteins. Following incubation and capacitation, sperm exhibited good levels of protein tyrosine phosphorylation and ATP levels with high T-AOC and CuZn-SOD activity and low MDA content. ZnO NPs exerted protective capacity to a preservation extender used for SP-free boar sperm during storage at 17 °C. The optimal concentration of ZnO NPs for preservation extender was 50 μg/mL.

## Introduction

Artificial insemination is commonly used in animal production and usually involves the dilution and preservation of semen (Prapaiwan et al., 2016). The main temperature for preservation is 17 °C because the fertilization rate is most similar to the natural fertilization rate at this temperature. However, the dilution and preservation of sperm are both processes that can be damaged by low temperature damage, oxidative stress and bacterial infection (Holst et al., 2001). The damage caused by ejaculation, dilution mechanical force and bacterial breeding during sperm preservation can lead to the accumulation of oxides. Moreover, the destruction of sperm’s structure and the reduction of its antioxidant enzyme activity can reduce the antioxidant capacity. Therefore, the destruction of the sperm antioxidant enzyme system and the surge of oxides lead to cellular oxidative stress (Lee and Park, 2015). Previous research has focused on the exogenous addition of antioxidants and bacteriostatic agents to reduce the damage incurred by sperm, while ensuring the physical environment of the sperm *in vitro* and maintaining normal sperm functionality (Nabeel et al., 2019). In particular, the porcine sperm membrane is vulnerable to low temperatures and oxidative stress, which has a low cholesterol/phospholipid ratio (Qamar et al., 2020).

Nanotechnology is an emerging field of biotechnology, and its application in a range of biomedical applications has been confirmed. Zinc oxide nanoparticles (ZnO NPs) are oxidizing particles with a particle size ranging from 1-100 nm that exhibits excellent properties (Rasmussen et al., 2010); in particular, these particles can protect sperm from the damage caused by freezing and thawing, especially DNA damage (Kumaran et al., 2016). Zinc oxide nanoparticles are widely used in animal production and is known for their high biological activity, good bactericidal effects and good safety profile. In the present study, the exogenous addition of antibacterial and antioxidant substances to ZnO NPs was applied to a porcine sperm diluent in an attempt to maintain the quality of preserved semen; we also analyzed the specific mechanisms involved.

The mature mammalian sperm is a highly differentiated germ cell that generates the capacity to fertilize after capacitation (Darves-Bornoz et al., 2021). Tyrosine phosphorylation is a very important link in the capacitation process and plays a role in the regulation of the acrosome reaction and the hyperactivation related to capacitation (Brohi and Huo, 2017). Investigating the molecular mechanisms of capacitation is helpful if we are to solve the problems associated with clinical male infertility and male contraception, and to provide theoretical support for the application of *in vitro* artificial insemination in livestock. During storage at 17 °C, the nutrients contained seminal plasma might induce bacterial and oxidative stress. In the present study, we analyzed a range of sperm quality parameters, including ATP content, antioxidant capacity, protein tyrosine phosphorylation, in order to understand the protective effects of ZnO NPs on the preservation of seminal plasma-free boar sperm at 17 °C preservation.

## Methods

### Media and experimental design

Sperm-rich fractions were collected from seven healthy adult Duroc boars (age range 1.5-2 years old) with good motility (at least 70% motile spermatozoa). In total, we collected sperm form seven Duroc boars every two weeks five consecutive times. Sperm was collected by the gloved-hand method applied by the technical staff of a local farm (Tianjin, China). Mixed the freshly collected boar semen with diluent in a volume of 1:2 and removed one-third of the supernatant by centrifugation (700 × *g*, 5min). Then, the diluted sperm were appropriately diluted with Androstar® Plus extender (Minitüb GmbH, Tiefenbach, Germany) supplemented with ZnO NPs (XFNANO Materials Tech Co Ltd., Nanjing, China) at 0 (control group), 20, 50, 100, or 200 μg/mL to achieve a final concentration of 1 × 10^7^ cells/mL (Fu et al., 2017). Nano-ZnO solutions were prepared in deionized water and dispersed by a probe-sonicator for 20 min before used. All sperm samples were stored at 17 °C for 7 days.

The basal medium was modified Whitten’s media (Moore et al., 1994). The non-capacitating (N-Cap) medium (pH 7.4) consisted of 2.7 mM KCl, 1.5 mM KH_2_PO_4_, 8.1 mM NaH_2_PO_4_, 137 mM NaCl, 5.55 mM Glucose and 2 mM Sodium pyruvate. The capacitating (Cap) medium (pH 7.4) consisted of 4.8 mM KCl, 1.2 mM KH_2_PO_4_, 95 mM NaCl, 5.55 Glucose, 25 mM NaHCO_3_, 2 mM CaCl_2_, 0.4% BSA and 2 mM Sodium pyruvate, as described by Tardif et al. (2001).

Non-capacitated sperm were collected using non-capacitating medium and incubated for 2 h at 37 °C (Tardif et al., 2003). Capacitated sperm were collected and then incubated with capacitated medium for 2 h at 37 °C (Zhen et al., 2016). The medium was shaken every 10 min to prevent the precipitation formed by incubation in capacitating conditions which can induce head-to-head agglutination. Then, we analyzed sperm motility, ATP level, T-AOC, CuZn-SOD activity, MDA content and (total, triton-soluble and triton-insoluble) protein tyrosine phosphorylation.

### The total sperm motility and plasma membrane integrity

The total sperm motility was determined in each group by using a pre-warmed sperm counting chamber (MAILANG, China) on a light microscope (OLYMPUS, CX21) fitted with a 37 °C thermostatic table. We used the iSperm^®^ to detected the non-capacitated sperm in progressive situations.

The integrity of the sperm membrane was assessed by staining with Coomassie Brilliant Blue. The protein component of the acrosome of sperm with an intact membrane was be dyed dark blue by Coomassie Brilliant Blue staining solution, while sperm with an incomplete membrane remained unstained. Approximately 200 sperm were examined under a light microscope.


$$Membrane\;integrity\;\left(\%\right)\;=\;\left(number\;of\;stained\;sperm\;/\;200\right)\;\times \;100$$ (1)

### Detection of ATP content

We used an ATP assay kit to determine the ATP content of sperm (Nanjing Jiancheng Bioengineering Institute, China) in accordance with the manufacturer’s instructions. Sperm sample was collected from each bottle, washed three time with doble tilled water (DDH_2_O) and then sonicated. Each sample was then boiled in the 100 °C water for 5 min. Supernatants were collected and mixed with the reaction buffer. Finally, a spectrophotometer was used to measure the ATP content at 636 nm.

### Antioxidant enzyme activity test

The CuZn-SOD activities were determined to assess the responses of the antioxidant defense system in sperm. For this, we used commercial kits (Nanjing Jiancheng Bioengineering Institute, China) in accordance with the manufacturer’s instructions. The semen samples were washed three times with PBS and then resuspended. The sperm sample used to analyze the CuZn-SOD activity were mixed with the kit butter, vortexed for 1 min and then centrifuged (12,000 × *g*, 5 min) to remove debris. Finally, the CuZn-SOD activity of each sample was measured with a spectrophotometer at a wavelength of 550 nm.

### Total antioxidant capacity activity assay

The total antioxidant capacity activity was quantified using a T-AOC assay kit (Nanjing Jiancheng Bioengineering Institute, China) in accordance with the manufacturer’s instructions. Each sperm supernatant was washed and resuspended in DDH_2_O, then by sonicated. The suspension was lysed on ice and then the sample was mixed with reaction buffer. Finally, the T-AOC activity of each sample was measured with a spectrophotometer at a wavelength of 520 nm.

### Malondialdehyde (MDA) content assay

Malondialdehyde content was determined by an MDA test kit (Nanjing Jiancheng Bioengineering Institute, China) in accordance with the manufacturer’s instructions. The sperm samples were collected and washed three times. Then, the resuspended samples were lysed ultrasonically on ice and centrifuged (12,000 × *g*, 5 min) to remove debris. Finally, a spectrophotometer was used to measure the MDA content at 532 nm.

## Protein immunoblotting

### Protein separation and quantification

#### Total-protein

After incubation, samples from each group were centrifuged with a low-temperature high-speed centrifuge (12,500 × *g*, 4 °C) for 5 min. We then collected the cell precipitate with cool phosphate buffer solution (PBS: 8 g NaCl, 0.2 g KH_2_PO_4_, 1.15 g Na_2_HPO_4_, 0.2 g KCl, DDH_2_O total to 1 L). Next, the supernatant was removed and the pellets were re-suspended in 200 μL of protein lysate (1.67 mL DDH_2_O, 0.5 mM pH 6.8 5.83 mL Tris-base, 2.5 mL Glycerol, 833 mg SDS, 100 μL protease inhibitors, total 10 mL), boiled for 4 min and then centrifuged at 12,500 × *g* for 15 min. Next, the supernatant was transferred to a new centrifuge tube, mixed with 10% β-mercaptoethanol and then boiled for 3 min. Subsequently, we used a Microplate Reader (Biorad, USA) and a Protein Quantitative Kit (Beyotime Institute of Biotechnology, Shanghai, China) to determine the concentration of sperm protein.

#### Triton-soluble and insoluble protein

Triton-insoluble and soluble protein were separated with 0.1% Triton X-100 buffer using a 30 min cycle of vortex mixing (5 min) and incubation in an ice-bath (5 min). The method used for protein lysis was the same as that for total-protein.

### SDS-PAGE and immunoblotting

Sperm proteins extracted from 1 × 10^6^/mL sperm and separated by SDS-PAGE using 10% polyacrylamide gels, as described by Tulsiani et al. (1995). Protein samples were separated by 12% acrylamide gel electrophoresis and then transferred to PVDF membranes using a transfer electrophoresis cell (10 V, 12 h). After blocking for 1 h with 1%BSA, the membranes were incubated with tyrosine phosphorylated antibody (Cat# 05-321, clone 4G10, Millipore, USA) for 4 h at 4 °C. The membranes were then washed three times with the washing buffer (T-TBS) for 15 min each time. Membranes were then incubated with the corresponding secondary antibody for 2 h at 4 °C. Membranes were then incubated with ECL reagent for 2 min. Excess ECL was then removed and the membranes were exposed to a film in a dark room. Films were developed, fixed, and then photographed with Bio-Rad gel imaging system (Bio-Rad, USA). Subsequently, the antibody was removed from the PVDF membranes, which were then re-probed with a β-actin antibody.

### Immunofluorescence

Following incubation, 300 μL of each sample was suspended in 10% formaldehyde solution and fixed for at least 12 h at 4 °C. The samples were then washed three times in PBS at low temperature, then evenly smeared onto a slide that was then allowed to air dry at room temperature for 2 h. The air-dried samples were then fixed with 3.7% formaldehyde for 20 min and permeabilized with 0.5% Triton X-100 in PBS for 10 min. The sperm were blocked with 1% BSA in PBS for 2 h, between each step, the slides were washed five times in PBS. The treated sperm samples were then incubated with tyrosine phosphorylated antibody (1:5000) overnight at 4 °C, and then washed five times with PBS (3 min per wash). Next, the slides were incubated with a fluorescent secondary antibody (1:1000) for 2 h at 4 °C. Finally, we added anti-fluorescence quenching agent and acquired photographs by fluorescence microscopy.

## Data analysis

All experiments were performed three times independently. The values of the control group and treatment groups were compared by analysis of variance (ANOVA) and Duncan’s multiple range tests using SPSS 20.0 software; *p* < 0.05 was considered statistically significant for all tests.

## Ethics approval and consent to participate

No ethical approval was required for this research because the animals were not produced for experimental purposes. The biological material was collected in semen for commercial boars belonging to the breeding farm. This article does not feature any data acquired from live animals; therefore, no ethics approvals were required.

## Results

### The effects of different concentrations of ZnO NPs on sperm motility and plasma membrane integrity

**Figure 1:**
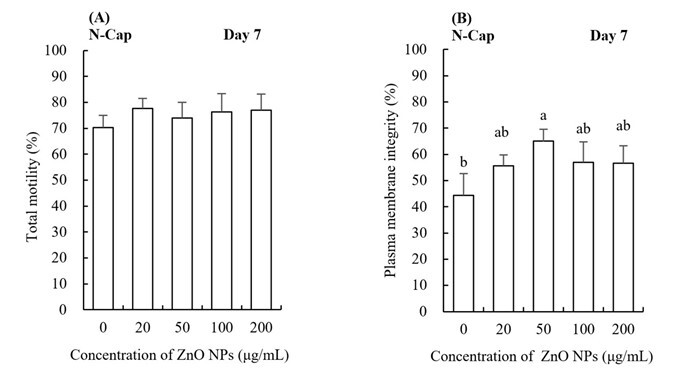
The effects of ZnO NPs on the total motility (**A**) and plasma membrane integrity (**B**) of boar semen preserved at 17 °C. The data are expressed as the mean ± SD. Different lowercase letters demonstrate significant differences (*p* < 0.05), whereas the same lowercase letters denote insignificant differences. N-Cap and Cap refer to sperm under respectively non-capacitating conditions and capacitating conditions.

The effects of different concentrations of ZnO NPs on boar semen motility during liquid preservation at 17 °C are shown in Figure 1A. The total motility in the ZnO NPs group was not significantly different from the control group in non-capacitating conditions.

The adding to ZnO NPs (50 μg/mL) led to a significant (*p* < 0.05) higher membrane integrity as compared to the control group (Figure 1B).

### The effects of ZnO NPs supplementation on the ATP content of boar sperm

**Figure 2:**
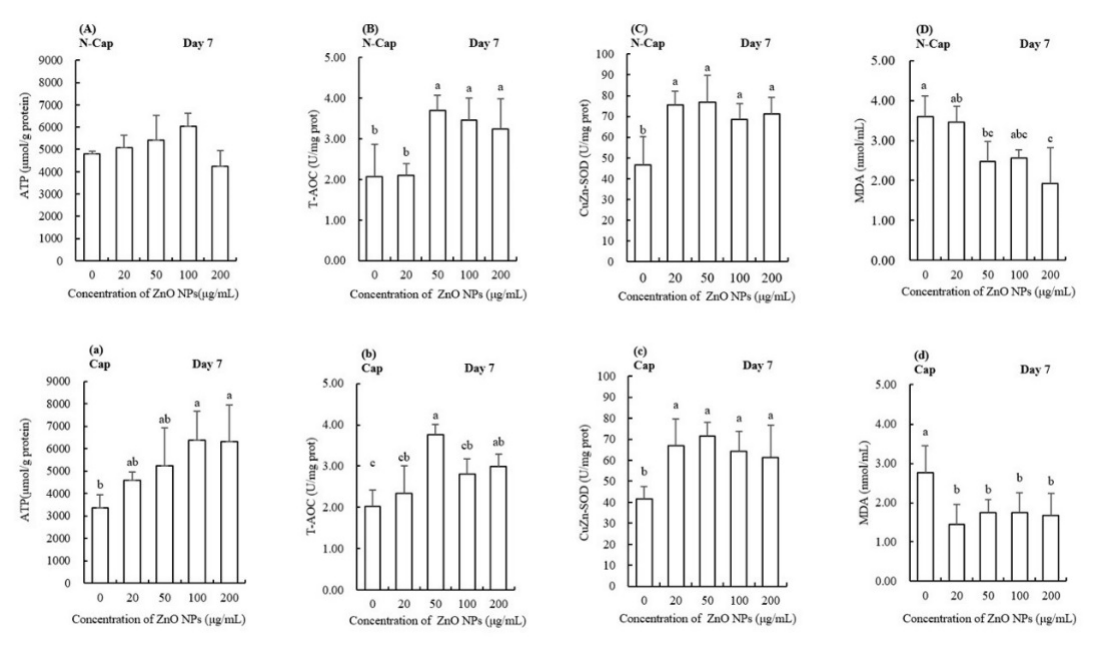
The effects of ZnO NPs on the ATP content (**A a**), T-AOC (**B b**), CuZn-SOD activity (**C c**) and MDA content (**D d**) of boar semen preserved at 17 °C for 7 days. The data are expressed as the mean ± SD. Different lowercase letters demonstrate significant differences (*p* < 0.05), whereas the same lowercase letters denote insignificant differences. N-Cap and Cap refer to sperm under respectively non-capacitating conditions and capacitating conditions.

The levels of ATP in boar sperm treated with ZnO NPs is shown in Figure 2(A, a). After 7 days of preservation, there was no significant difference in ATP content when compared between the control group and groups that had been supplemented with ATP (*p* < 0.05) in non-capacitating conditions. However, in capacitating conditions, the groups exposed to 100 and 200 μg/mL ZnO NPs had significantly higher levels of ATP (*p* < 0.05). The untreated group had the lowest levels of ATP of all the groups in both non-capacitated and capacitated conditions.

### The effect of ZnO NPs on boar sperm antioxidation ability

To verify the potential protective effects of ZnO NPs in antioxidative and oxidative states, we measured the T-AOC, CuZn-SOD activities, and MDA content. As shown in Figure 2(B, b-D, d), when compared with the control group, T-AOC activity in the groups treated with 50, 100 and 200 μg/mL ZnO NPs had higher T-AOC activity (*p* < 0.05).

Zinc ions are a key component of the CuZn-SOD enzyme; therefore, we investigated the activity of this enzyme to understand the effect of ZnO NPs on the antioxidant system in sperm. As shown in Figure 2(C, c), ZnO NPs had a significant effect on the activity of CuZn-SOD; activity was higher in all treatment groups compared with the control group (*p* < 0.05); this was the case for both the capacitating and non-capacitating conditions.

Sperm MDA in groups supplemented with (50,100 and 200 μg/mL) ZnO NPs was significantly lower than the control group, both in the non-capacitated and capacitated conditions, as shown in Figure 2(D, d) (*p* < 0.05).

### The effect of ZnO NPs on the tyrosine phosphorylation

**Figure 3:**
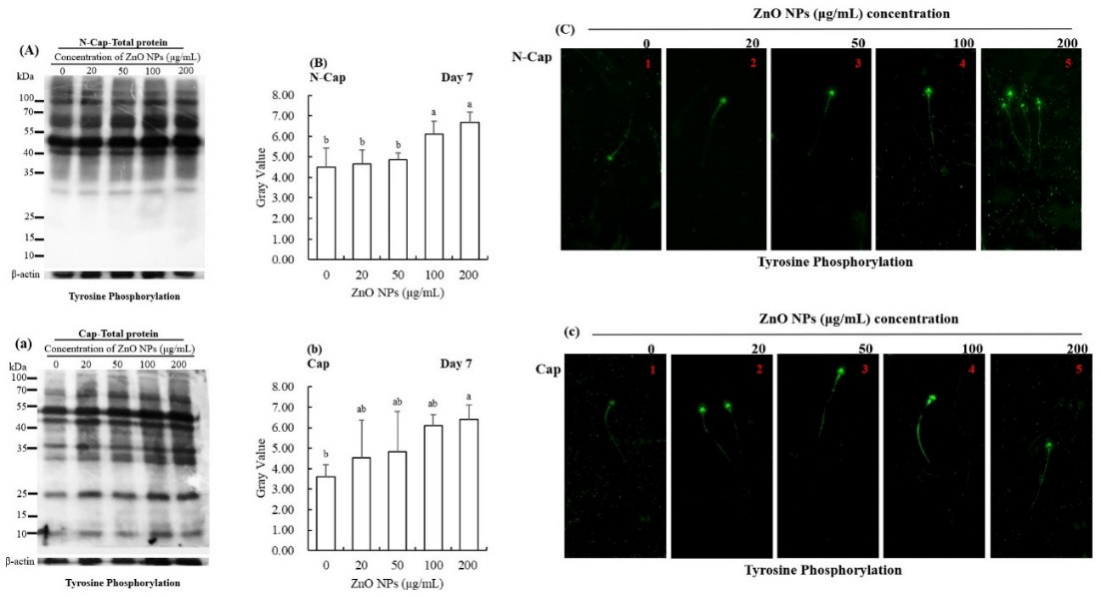
Western blot analysis of PTP on total protein when exposed to different concentrations of ZnO NPs. Western blot analysis was performed using an anti-phosphotyrosine antibody (**A, a, C, c**) and (**B, b**) is protein gray value. β-actin was used as an internal control. The experiment was performed in triplicate (n = 3, *p* < 0.05). Immunolocalization of tyrosine-phosphorylated protein in boar sperm stored at 17 °C with different concentrations of ZnO NPs (**C, c**). N-Cap and Cap refer to sperm under respectively non-capacitating conditions and capacitating conditions.

The level of PTP was greatly higher in the treatment groups than that in the control group (Figure 3B and b, *p* < 0.05). The total protein PTP was increased by treatment with ZnO NPs, particularly at 100 and 200 μg/mL. After incubation in capacitating conditions, the total protein PTP in the 200 μg/mL ZnO NPs supplement group was significantly increased (Figure 3b, *p* < 0.05). Furthermore, the immunolocalization of demonstrated the enhancement of protein tyrosine phosphorylation on the principle-pieces of the flagella in response to ZnO NPs, as demonstrated by western blotting results (Figure 3C and 3c).

**Figure 4:**
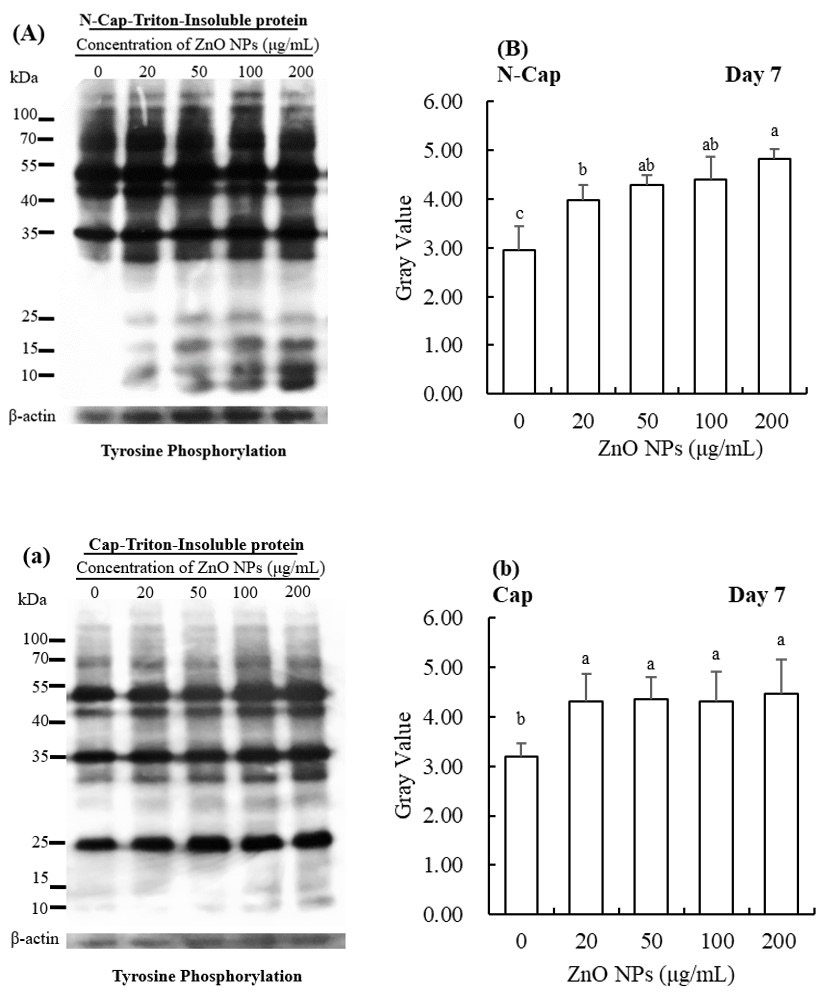
Western blot analysis of PTP on triton-insoluble protein when exposed to different concentrations of ZnO NPs. Western blot analysis was performed using an anti-phosphotyrosine antibody (**A, a**) and (**B, b**) is protein gray value. β-actin was used as an internal control. The experiment was performed in triplicate (n = 3, *p* < 0.05). N-Cap and Cap refer to sperm under respectively non-capacitating conditions and capacitating conditions.

**Figure 5:**
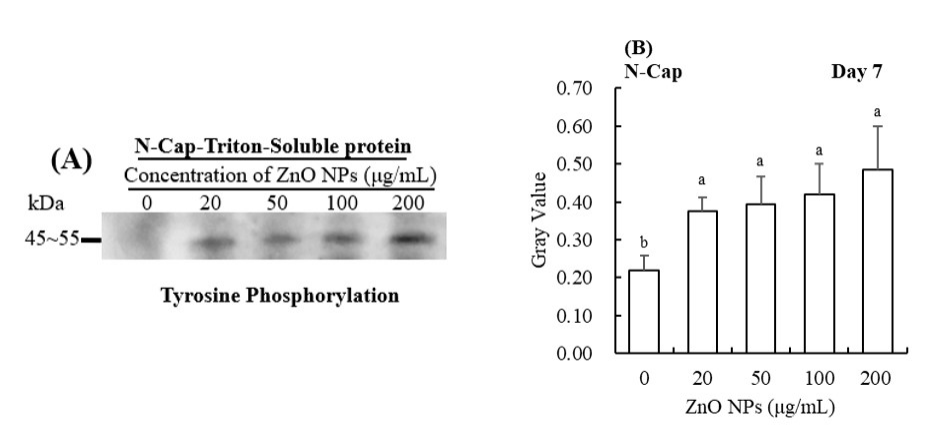
Western blot analysis of PTP on triton-soluble protein when exposed to different concentrations of ZnO NPs. Western blot analysis was performed using an anti-phosphotyrosine antibody (**A**) and (**B**) is protein gray value. β-actin was used as an internal control. The experiment was performed in triplicate (n = 3, *p* < 0.05). N-Cap refer to sperm under non-capacitating conditions.

The changes of PTP on triton-insoluble proteins followed the same trend as with total protein; the ZnO NPs had a more significant effect than in the control group (*p* < 0.05, Figure 4), both in non-capacitated or capacitated conditions. Similarly, the triton-soluble protein PTP in ZnO NPs groups were significantly higher than in the control group (*p* < 0.05, Figure 5).

## Discussion

Because of the plasma membrane structure of boar, the semen preservation temperature prefers 17 °C rather than freezing (Zhang et al., 2017). Mammalian seminal plasma plays an important role in providing an appropriate living environment (osmotic pressure and pH) and energy for isolated sperm, reducing mechanical damage to sperm during ejaculation, providing antioxidant enzymes and avoiding sperm hyperactivation (Lamirande and Gagnon, 1995). However, when sperm is stored at room temperature, it is easy to cause bacterial breeding and oxidative accumulation perhaps due to sugars, protein and lipids in semen, which reduces the sperm storage time and sperm quality. So, during semen preservation processing, seminal plasma is removed and substituted with extender media. The ZnO NPs were efficient (green) antioxidants with special physical and chemical characteristics as a potential additive of exogenous diluent for 17 °C storaged with seminal plasma-free boar semen.

The seminal plasma contains millimolar concentration of Zn ions (Marzec-Wróblewska et al., 2011). Zinc ion in seminal plasma plays an important role and is a component of copper-zinc superoxide dismutase enzyme (Juyena and Stelletta, 2012). The concentration of antioxidants in seminal fluid is higher than that in any other biological fluid. CuZnSOD is one of the main enzymes in the semen that protects sperm from the excessive production of ROS (Feng et al., 2020), which also plays an important role in preventing hyperactivation (Britto et al., 2018). In the present study, the addition of ZnO NPs increased the CuZn-SOD activity of sperm under both non-capacitation and capacitation conditions (*p* <0.05).

Over the last decade, studies have indicated that ZnO NPs, due to the physicochemical properties of their large surface area, are able to scavenge ROS to protect cell membrane integrity from oxidative damage. In particular, it is worth noting that ZnO NPs have been demonstrated to reduce MDA-induced oxidative stress (González-Abreu et al., 2017). In the present study, we found that the sperm T-AOC activity were improved and the MDA content reduced, especially in the 50 μg/mL of ZnO NPs (*p* < 0.05) during preservation. The reasons for the improvement in total antioxidant capacity may be associated with the high-quality sperm membrane integrity.

In the ejaculated boar semen and semen preserved at room temperature, there is a significant reduction in sperm motility due to a lack of oxygen. Generally, the preservation of semen involves the use of an acidic environment to inhibit the movement of sperm, reduce energy consumption, and keep the sperm in a reversible static state without losing the ability to fertilize. In semen samples preserved with the addition of ZnO NPs, spermatozoa motility and ATP content did not differ significantly from the control groups (*p* > 0.05). When the motility is too high, it can cause the loss of nutrients and the accumulation of oxidation products; this is not conducive to preservation.

We found that supplementation with ZnO NPs had the protective effect on protein tyrosine phosphorylation, which was mainly located in the middle and principal-pieces of sperm flagellum. Triton-insoluble and triton soluble protein tyrosine phosphorylation also increased with the administration of ZnO NPs in a dose-dependent manner. Triton insoluble protein contains nucleoprotein and skeleton proteins while the triton soluble protein included membrane proteins. Protein tyrosine phosphorylation is involved in regulating sperm function, including energy metabolism. Mitochondria exist in the middle segment of sperm, providing sperm vitality through the oxidative phosphorylation pathway.

The zinc ions in prostate fluid can maintain the sperm chromatin and ODF structure in a soft state and avoid mechanical damage during ejaculation (Björndahl and Kvist, 2010). Zinc ions also improved DNA methylation and chromatin integrity (Quinn, 1968). It is speculated that ZnO NPs have a protective effect on the preservation of sperm nucleoprotein. Parts of sperm zincoproteins are cytoskeletal proteins. (Eagle et al.,1983). It is speculated that ZnO NPs can protect sperm skeleton proteins.

Spermatozoa interact with and are altered by their immediate environment, and the plasma membrane serves as the two-way communication device. Sperm surface changes and physiological consequences induced by storage (Leahy and Gadella, 2011). The long-term storage of sperm needs to consider the fact that boar sperm have a fragile and sensitive structure with a low cholesterol/phospholipid ratio (Naresh and Atreja, 2015). The structural integrity of sperm is responsible for maintaining full sperm functionality (Lee and Park, 2015). In the present study, we found that ZnO NPs can protect the integrity of the boar sperm membrane, especially in the 0.5 μg/mL concentration (*p* < 0.05). Maintaining the integrity of the sperm acrosome is a key step to ensure that sperm complete a series of complex fertilization processes (Yeste et al., 2017). The functional state of the membrane proteins can be directly confirmed by analyzing the phosphorylation state of the sperm membrane protein.

Prior to fertilization, sperm undergo capacitation in the female reproductive tract. Protein tyrosine phosphorylation and actin polymerization are two important processes involved in the capacitation of mammalian sperm (Brener et al., 2003). During sperm capacitation, actin polymerizes in the tail region and then progresses to the head region; tyrosine phosphorylation is known to regulate actin polymerization (Cabello-Agüeros et al., 2003). The localization of actin between the plasma membrane and the outer acrosome membrane in the sperm of several mammals indicates that this protein plays a key role in sperm capacitation and the acrosome reaction (Breitbart et al., 2005). The cytotoxic effects of ZnO NPs are also the focus of attention. Therefore, it is judged that this dose of ZnO NPs has no toxic effect on sperm by analyzing capacitated sperm model. By analyzing sperm tyrosine phosphorylation, we found that ZnO NPs did not interfere with protein phosphorylation on membrane or cytoskeleton.

Therefore, the protection of sperm against oxidative stress by antioxidant supplementation is currently a significant research topic. Importantly, these discoveries contribute to a more comprehensive view of the molecular mechanisms underlying the protective effects of exogenous antioxidants on sperm; collectively, the present findings indicate that ZnO NPs are safe and practical to use as a supplement for boar semen extenders to facilitate assisted reproductive technology.

## Conclusion

It was speculated that ZnO NPs could effectively protect the viability of boar spermatozoa by improving the antioxidant capacity; And by eliminating excessive malondialdehyde, it can effectively protect sperm protein phosphorylation modification, especially membrane protein, and improved capacitation related functions. Our results suggested that ZnO NPs could effectively protect boar sperm against oxidative injury. This approach can represents a good alternative to the preservation methods used for boar semen. In summary, the supplementation of 50 μg/mL of ZnO NPs was able to maintain sperm quality during liquid preservation at 17 °C under seminal plasma-free conditions.
